# Supplementary material for: The actin cytoskeletal architecture of estrogen receptor positive breast cancer cells suppresses invasion
Source: Nat Commun. 2018 Jul 30;9:2980. doi: 10.1038/s41467-018-05367-2 (PMC6065369; doi:10.1038/s41467-018-05367-2)
Supplement: Supplementary file 1 — Supplementary Information [file 41467_2018_5367_MOESM1_ESM.pdf]

SUPPLEMENTARY INFORMATION

Estrogen Receptor Suppresses Invasion by EVL Mediated Actin Cytoskeletal Remodeling.

Marco Padilla-Rodriguez, Sara S. Parker, Deanna G. Adams, Thomas Westerling, Julieann Puleo, Adam W. Watson, Samantha M. Hill, Muhammad Noon, Raphael Gaudin, Jesse Aaron, Daoqin Tong, Denise J. Roe, Beatrice Knudsen and Ghassan Mouneimne.

a

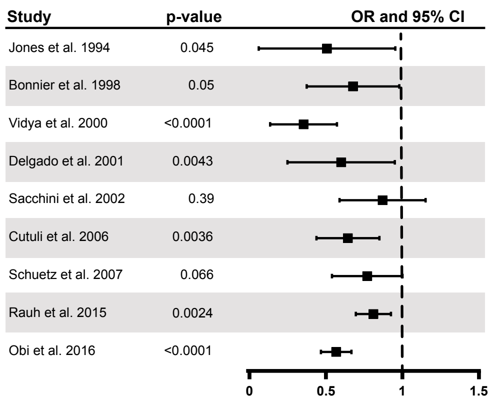

b

Study		Never-Users	Current-Users
Jones et al. 1994	LN -	106	23
	LN +	82	7
Bonnier et al. 1998	LN -	146	89
	LN +	116	85
Vidya et al. 2000	LN -	103	40
	LN +	148	18
Delgado et al. 2001	LN -	63	76
	LN +	45	29
Sacchini et al. 2002	LN -	477	120
	LN +	417	91
Cutuli et al. 2006	LN -	218	170
	LN +	202	99
Schuetz et al. 2007	LN -	265	201
	LN +	208	119
Rauh et al. 2015	LN -	1953	931
	LN +	1159	449
Obi et al. 2016	LN -	636	1058
	LN +	444	418

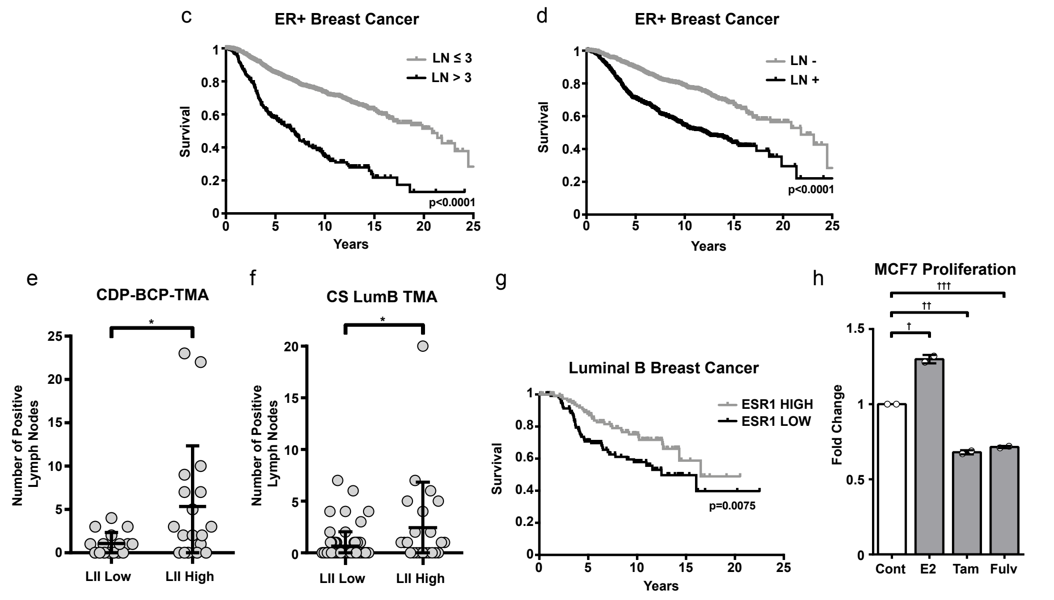

Supplementary Fig. 1 (related to Fig.1).

(a) Binary Assessment of LN Dissemination (LN+ vs LN-). Forest plot showing Odds Ratio, with 95% Confidence Interval (OR, 95% CI) of LN positivity in current-users compared with never-users of HRT. **(b)** Number of current-users and never-users with either LN- or LN+ tumors in the studies analyzed in (a). **(c)** Kaplan-Meier plot showing survival of ER+ breast cancer patients clustered by LN status as LN≤3 and LN>3; $p<0.001$ (Chi-square test). ER levels are 1.8X higher in LN≤3. Data are from Curtis et al. (1651 patients in LN≤3 group, 314 in LN>3 group). **(d)** Kaplan-Meier plot showing survival of ER+ breast cancer patients clustered by LN status as LN- and LN+; $p<0.001$ (Chi-square test). ER levels are 1.5X higher in LN+. Data are from Curtis et al. (1032 patients in LN- group, 933 patients in LN+ group). **(e)** Scatter plot showing quantification of positive LN in Low (≤7μm) and High (≥9μm) LII tumors from NCI BCP TMA (TMA#1); values are mean±s.d.; *p=0.03 (Mann-Whitney exact t-test). **(f)** Scatter plot showing quantification of positive LN in tumors with Low (≤7μm) and High (≥9μm) LII tumors from Cedar-Sinai LumB TMA (TMA#2); values are mean±s.d.; *p=0.01 (Mann-Whitney exact t-test). **(g)** Kaplan-Meier plot showing survival of Luminal B ER+ breast cancer patients clustered by ESR1 expression as ESR1 HIGH (3rd quartile) and ESR1 LOW (1st quartile); $p<0.05$ (Chi-square test). Data are from Curtis et al. (123 patients in ESR1 HIGH group, 123 patients in ESR1 LOW group). **(h)** Quantification of proliferation of MCF7 cells treated with respective drugs for 48 hours. Fold change in proliferation is shown between treatments. Data pooled from two independent experiments; mean±s.d. †p=0.02, ††p=0.04, †††p=0.01 (Welch's t-test).

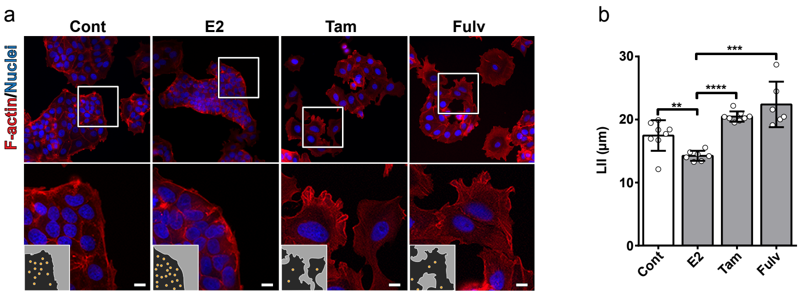

Supplementary Fig. 2 (related to Fig.2).

(a) F-actin staining of MCF7 cells after corresponding treatments. Lower panels are magnifications of boxed areas. Insets are binary masks of actin stain (black) and nuclei (orange). Scale bar is 10μm. **(b)** Quantification of LII across treatment groups. Data are from two independent experiments, n>400 cells per treatment; mean±s.d. **p=0.006, ***p=0.002, ****p<0.0001 (Welch's t-test).

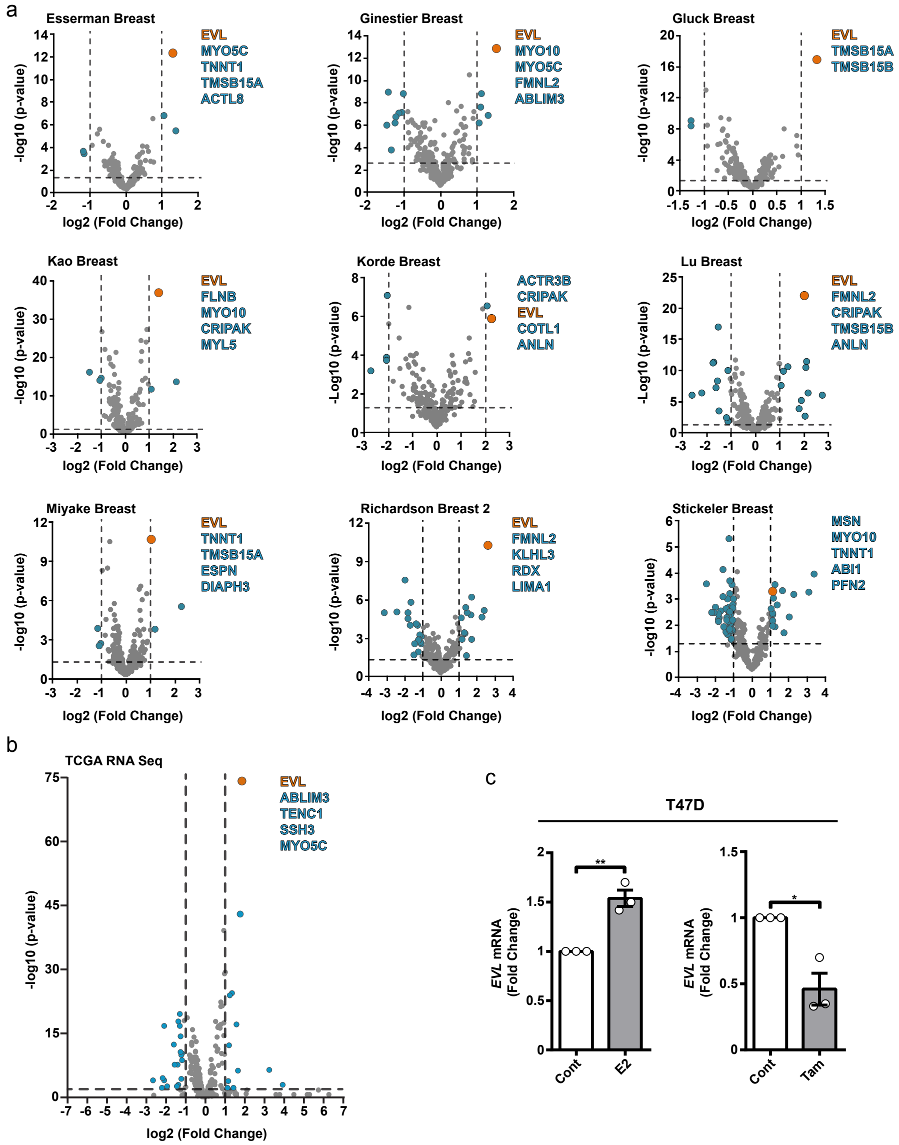

Supplementary Fig. 3 (related to Fig.4).

(a) Differential gene expression analysis of actin cytoskeletal regulators in ER+ vs. ER- breast tumors. Volcano plot of significance (-log10 of p-value) vs. log2 fold change. Horizontal dashed line represents threshold for significance at p<0.05. Vertical dashed lines represent threshold for positive or negative two-fold change in gene expression. Insets are lists of genes that passed both thresholds in descending order of significance (lists were limited to five genes; full data analyses are presented in Supplementary Data 3) **(b)** Volcano plot showing differential transcript levels between ER+ and ER- tumors using the TCGA RNA-seq data set (full data analysis is presented in Supplementary Data 4). **(c)** qPCR of *EVL* in T47D cells 24 hours post-treat-ment, normalized to *GAPDH*. Brackets show fold change between treatment groups. Data are from three independent experiments; mean±s.e.m. **p=0.007, *p=0.01 (Unpaired t-test).

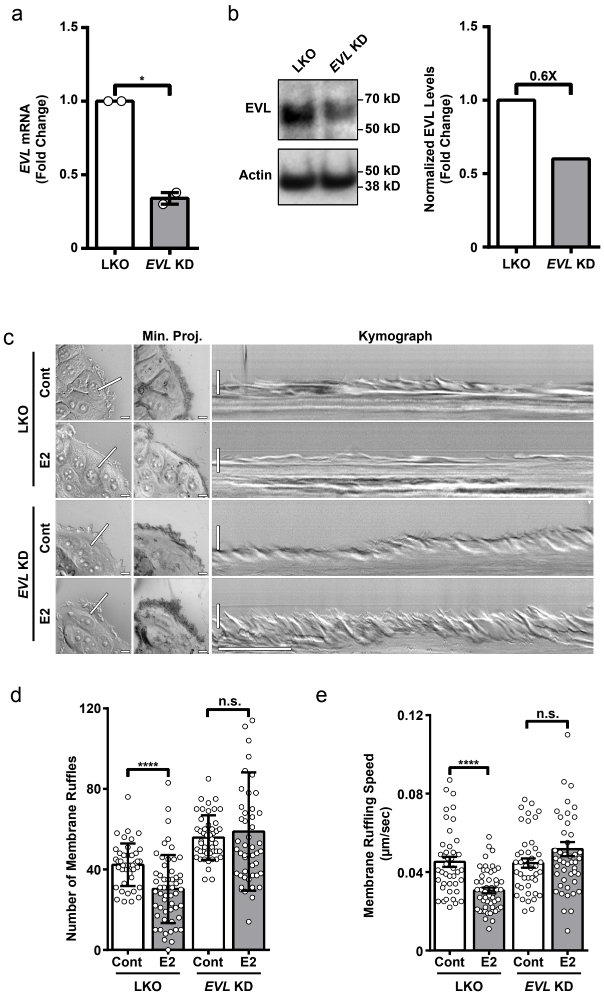

Supplementary Fig. 4 (related to Fig.5).

(a) qPCR of EVL mRNA from LKO and *EVL* KD MCF7 cells, normalized to *GAPDH*. Values are means of data from two independent experiments; mean±s.e.m. (Welch's t-test). **(b)** Left panel. Western blot of LKO and *EVL* KD MCF7 cell lysates showing EVL levels and actin loading control. Right panel. Quantification of western blot showing fold change in EVL levels, normalized to actin. **(c)** Leading edge kymography in representative time-lapse movies of LKO and *EVL* KD in MCF7 cells treated with vehicle or with E2 for 72 hours (Supplementary Movie 6). Left panels indicate position at which kymographs were registered (line), and middle panels show minimum intensity projections from the entire time series (Min. Proj.); scale bar is 10μm. Right panels show corresponding kymographs; vertical scale bar is 10μm; horizontal scale bar is 5 min. **(d)** Membrane ruffle quantification. Data are from three independent experiments, n≥45 per treatment group; mean±s.d. ****p<0.0001, n.s.=not significant (Unpaired t-test). **(e)** Ruffling speed quantification. Data are from three independent experiments,n≥45 per treatment group; mean±s.e.m. ****p<0.0001, n.s.=not significant (Unpaired t-test).

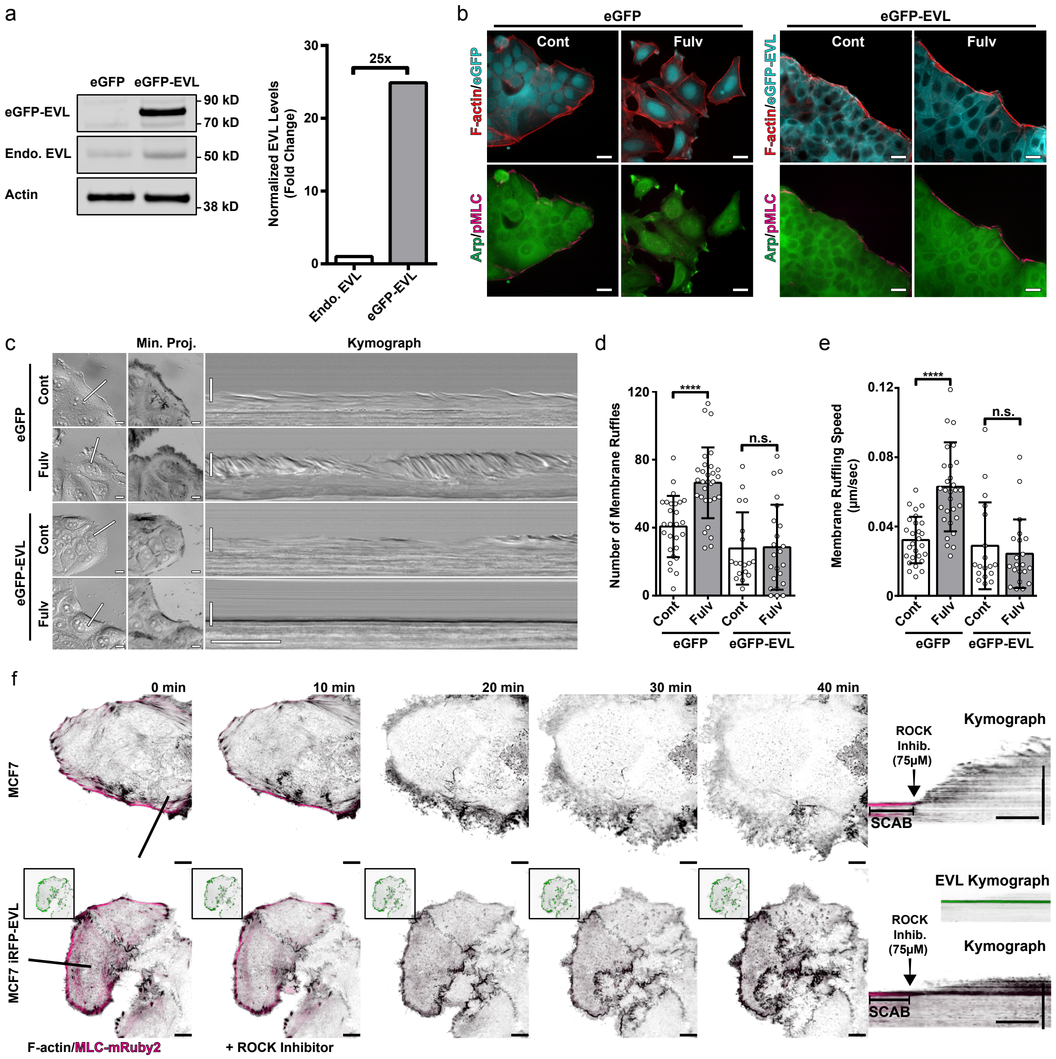

Supplementary Fig. 5 (related to Fig.5).

(a) Left panel. Western blot of eGFP and eGFP-EVL MCF7 cell lysates showing EVL levels and actin loading control. Right panel. Quantification of western blot showing fold change in EVL levels relative to endogenous EVL, normalized to actin. **(b)** Large composite stitched images of treatment groups in Fig.5c. Scale bar is 20μm. **(c)** Leading edge kymography in representative time-lapse movies of eGFP and eGFP-EVL in MCF7 cells treated with vehicle or with fulv for 72 hours (Supplementary Movie 7). Left panels indicate position at which kymographs were registered (line), and middle panels show minimum intensity projections from the entire time series (Min. Proj.); scale bar is 10μm. Right panels show corresponding kymographs; vertical scale bar is 10μm; horizontal scale bar is 5 min. **(d)** Membrane ruffle quantification. Data are from two independent experiments, n≥18 per treatment group; mean±s.d. ****p<0.0001, n.s.=not significant (Unpaired t-test). **(e)** Ruffling speed quantification. Data are from two independent experiments, n≥18 per treatment group; mean±s.e.m. ****p<0.0001, n.s.=not significant (Unpaired t-test). **(f)** Leading edge kymography in control and iRFP670-EVL (green) expressing MCF7 cells (top and bottom rows, respectively), with eGFP-Lifeact (black) and MLC-mRuby2 (magenta), before and after treatment with 75μM ROCK inhibitor (Supplementary Movie 9). Left panels are images from time-lapse series before and after treatment. Scale bar is 10μm. Line shows the leading edge location at which kymographs were registered. Inset shows EVL channel separately. Right panel shows kymograph. Vertical scale bar is 10μm and horizontal scale bar is 10 min.

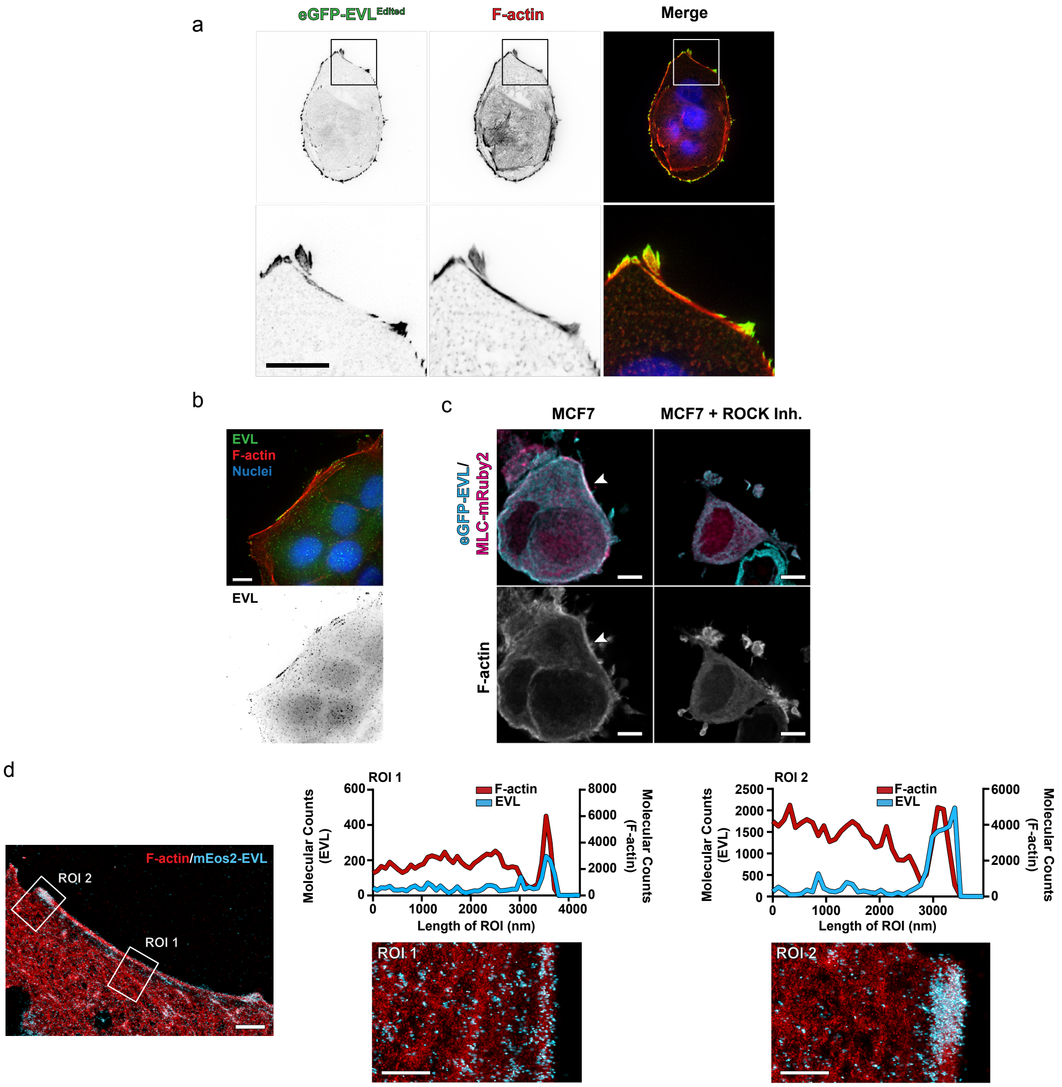

Supplementary Fig. 6 (related to Fig.5).

(a) TIRF microscopy images of eGFP-EVL^{edited} MCF7 cells. Lower panel are magnifications of boxed areas. Scale bar is 10µm. **(b)** Immunolabeling of EVL in MCF7 cells. Scale bar is 10µm. **(c)** Maximum projections of laser scanning confocal z-series of control and ROCK inhibitor-treated (25µM) MCF7 cells expressing eGFP-EVL (cyan) and MLC-mRuby2 (magenta) embedded in collagen. Arrows indicate a SCAB. Scale bar is 5µm. **(d)** Analysis of EVL localization at SCABs by iPALM of MCF7 cells stained for F-actin and expressing mEos2-EVL. Boxes indicate ROIs. ROIs shown with corresponding histograms plotting the molecular counts of F-actin and mEos2-EVL. Scale bar for merged image is 5µm. Scale bar for ROIs is 1µm.

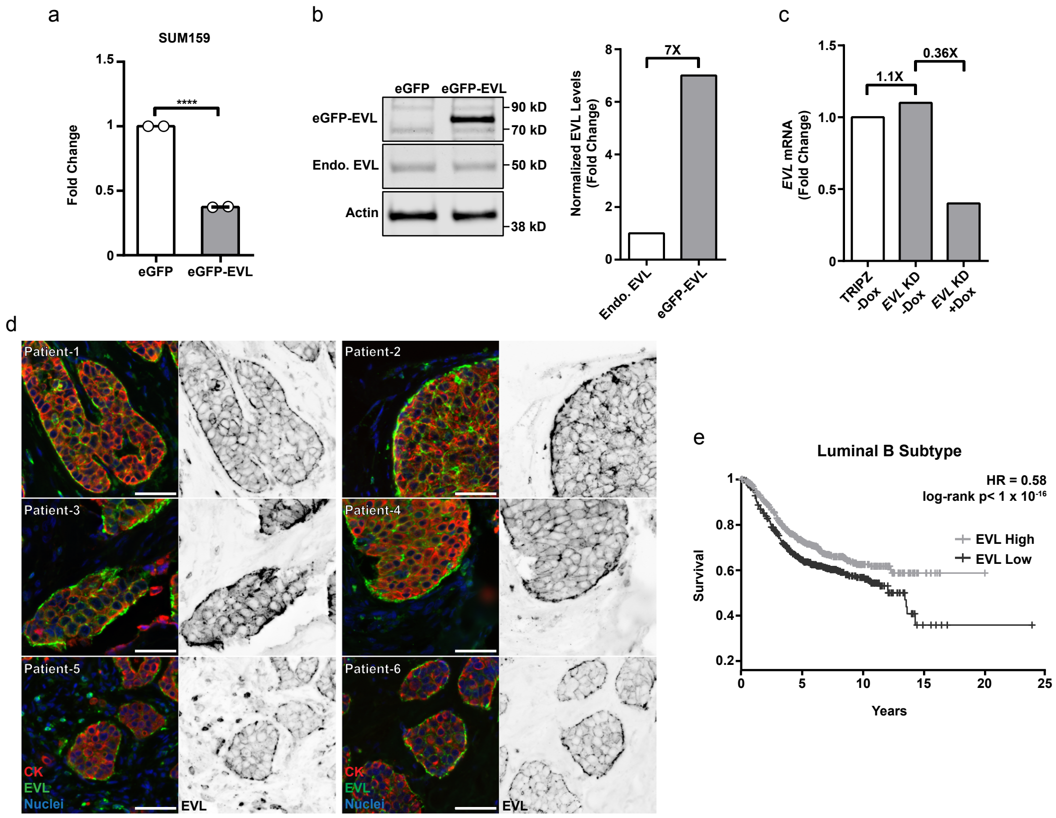

Supplementary Fig. 7 (related to Fig.6).

(a) Quantification of *in vitro* invasion of control and eGFP-EVL overexpressing SUM159 cells. Data are from two independent experiments, mean±s.d.; ****p<0.0001 (Welch's t-test). **(b)** Left panel. Western blot of eGFP and eGFP-EVL SUM159 cell lysates showing EVL levels and actin loading control. Right panel. Quantification of western blot showing fold change in EVL levels relative to endogenous EVL, normalized to actin. **(c)** qPCR of *EVL* mRNA in control TRIPZ MCF7 cells and inducible *EVL* KD with or without doxycycline (dox) induction. Brackets show fold change. **(d)** Representative images from six patient samples showing SCABs in tumors, as demarcated by EVL immunofluorescence labeling. Merged images show human cytokeratin in red, EVL in green, and nuclei in blue; single channels show inverted EVL. Scale bar is 50µm. **(e)** Kaplan-Meier plot showing survival of luminal B breast cancer patients clustered by EVL expression (split by at median). Data from KM Plotter (kmplot.com; Affy ID=217838_s_at/gene symbol=EVL). Hazard Ratio (HR) and log-rank p-value shown in inset (log-rank test); (575 patients in EVL High group, 574 in EVL Low group).

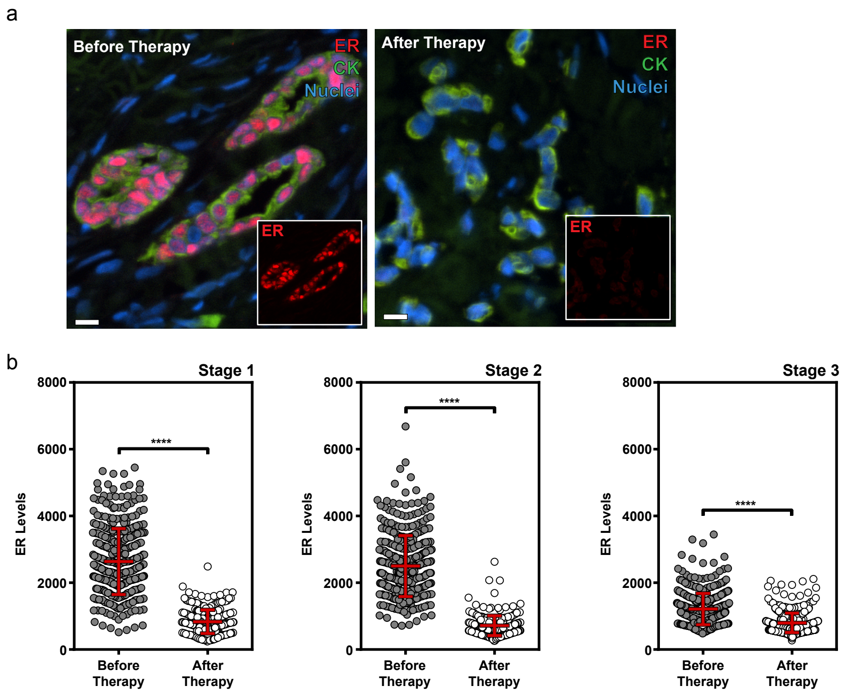

Supplementary Fig. 8 (related to Fig.7).

(a) Representative images of ER+ breast tumor before (left panel) and after (right panel) neo-adjuvant hormone therapy, immunolabeled for human cytokeratin shown in green, ER in red, and nuclei in blue. Scale bar is 10μm. Insets show ER channel separately. **(b)** Quantification of ER levels before and after hormone therapy. Scatter plots show the full range of cells analyzed within each tumor set: stage 1, n=389 cells before and n=295 cells after therapy; stage 2, n=372 cells before and n=265 cells after therapy; and stage 3, n=427 before and n=525 after therapy. Red lines represent means±s.d. ****$p<0.0001$ (Welch's t-test).

a

Supplementary Figure 4b

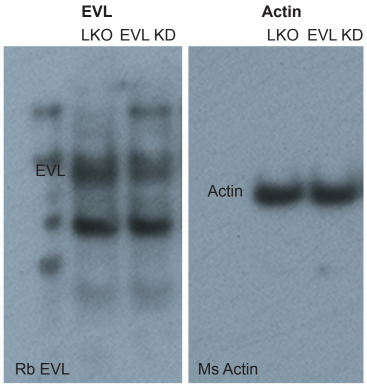

b

Supplementary Figure 5a

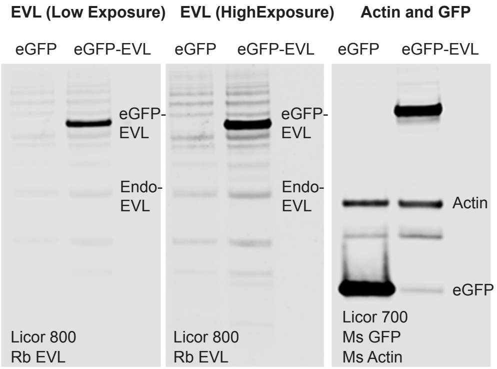

c

Supplementary Figure 7b

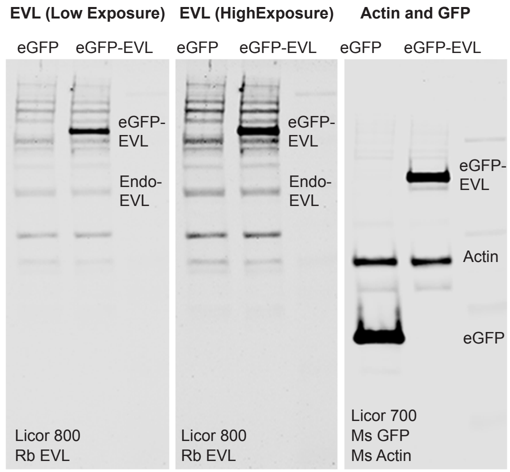

Supplementary Fig. 9

(a) Western blot of lysates from MCF7 cells expressing pLKO (left) or *EVL* shRNA (right). Membrane was probed with rabbit anti-EVL (a gift from F. Gertler) followed by goat anti-rabbit HRP secondary antibody (Thermo-Fisher). After obtaining film exposure, membrane was stripped and re-probed with mouse anti-actin (Abcam ab3280) followed by goat anti-mouse HRP antibody (Thermo-Fisher). **(b)** Western blot of lysates from MCF7 cells overexpressing either eGFP (left) or eGFP-EVL (right). Membrane was probed with rabbit anti-EVL (Sigma Prestige HPA018849), followed by anti-rabbit 800 (Licor) infrared secondary antibody. Membrane was scanned and re-probed with mouse anti-actin (ProteinTech 66009-1-Ig), and mouse anti-GFP (ProteinTech 50430-2-AP), followed by anti-mouse 680 infrared secondary antibody (Licor). **(c)** Western blot of lysates from SUM159 cells overexpressing either eGFP (left) or eGFP-EVL (right). Membrane was probed with rabbit anti-EVL (Sigma Prestige HPA018849) followed by anti-rabbit 800 infrared secondary antibody (Licor). Membrane was scanned and re-probed with mouse anti-actin (ProteinTech 66009-1-Ig), and mouse anti-GFP (ProteinTech 50430-2-AP), followed by anti-mouse 680 infrared secondary antibody (Licor).
