# Supplementary material for: The actin cytoskeletal architecture of estrogen receptor positive breast cancer cells suppresses invasion
Source: Nat Commun. 2018 Jul 30;9:2980. doi: 10.1038/s41467-018-05367-2 (PMC6065369; doi:10.1038/s41467-018-05367-2)
Supplement: Supplementary file 3 — Description of Additional Supplementary Files [file 41467_2018_5367_MOESM3_ESM.pdf]

Description of Additional Supplementary Files

File Name: Supplementary Data 1
Description: Curated list of actin regulators used in the analysis of differential gene expression.

File Name: Supplementary Data 2
Description: List of Microarray data sets used in the analysis of differential gene expression.

File Name: Supplementary Data 3
Description: Analysis of differential gene expression of curated list of actin regulators using microarray datasets.

File Name: Supplementary Data 4
Description: Gene expression analysis of curated actin regulators using Breast Cancer TCGA RNA-seq data set.

File Name: Supplementary Data 5
Description: Heat map showing relative expression of ESR1 and actin regulators.

File Name: Supplementary Data 6
Description: Correlation analysis between expression of ESR1 and actin regulators.

File Name: Supplementary Data 7
Description: ER-ChIP qPCR validation primer sequences.

File Name: Supplementary Data 8
Description: Clinical information on patient samples (each set includes two samples collected before and after treatment with hormone therapy).

File Name: Supplementary Data 9
Description: Primer sequences used in the different qPCR experiments in the manuscript.

File Name: Supplementary Movie 1
Description: Time-lapse Movie of MCF7 Cells Treated with E2 and ER Inhibitors. DIC images were acquired at 1 frame/sec. Scale bar is 10µm. WM is “white media” (phenol red-free media supplemented with charcoal-stripped FBS) and RM is “red media” (phenol red containing media supplemented with FBS).

File Name: Supplementary Movie 2
Description: . Time-lapse Movie of T47D Cells Treated with E2 and ER Inhibitor. DIC images were acquired at 1 frame/sec. Scale bar is 10µm.

File Name: Supplementary Movie 3
Description: Time-Lapse Movie of MCF7 cells Treated with E2 and ER Inhibitor. Widefield fluorescence microscopy of MCF7 cells expressing eGFP-Lifeact (cyan) were acquired at 1 frame/5min. Scale bar is 10µm.

File Name: Supplementary Movie 4

Description: Three-Dimensional Reconstruction of Actin Cytoskeleton in MCF7 Cells Treated with E2 and ER Inhibitor. 3D reconstruction of confocal z-series showing F-actin, pMLC, Arp2/3 and 3D volumetric actin mask.

File Name: Supplementary Movie 5
Description: Time-lapse Movie of SCAB Dissolution by ROCK inhibitor. TIRF microscopy time-lapse movie acquired at 1 frame/20 sec of MCF7 cells and T47D cells expressing iRFP-Lifeact (black) and MLC-mRuby2 (magenta) before and after treatment with 25µM ROCK inhibitor (added at time=10 min). Scale bar is 10µm

File Name: Supplementary Movie 6
Description: Time-lapse Movie of MCF7 LKO and EVL KD Cells Treated with E2. DIC images were acquired at 1 frame/sec. Scale bar is 10µm.

File Name: Supplementary Movie 7
Description: Time-lapse Movie of MCF7 eGFP and eGFP-EVL Cells Treated with ER Inhibitors. DIC images were acquired at 1 frame/sec. Scale bar is 10µm.

File Name: Supplementary Movie 8
Description: Time-lapse Movie of control and EVL-overexpressing MCF7 cells treated with 50µM ROCK inhibitor. TIRF microscopy time-lapse movies were acquired at 1 frame/20sec. Cells expressed iRFP-Lifeact (black) and MLC-mRuby2 (magenta), with or without EVL (green, shown separately in inset). ROCK inhibitor (50µM) was added at time=10 min. Scale bar is 10µm.

File Name: Supplementary Movie 9
Description: Time-lapse Movie of control and EVL-overexpressing MCF7 cells treated with 75µM ROCK inhibitor. TIRF microscopy time-lapse movies were acquired at 1 frame/20sec. Cells expressed iRFP-Lifeact (black) and MLC-mRuby2 (magenta), with or without EVL (green, shown separately in inset). ROCK inhibitor (75µM) was added at time=10 min. Scale bar is 10µm.
